# Supplementary material for: scBubbletree: computational approach for visualization of single cell RNA-seq data
Source: BMC Bioinformatics. 2024 Sep 13;25:302. doi: 10.1186/s12859-024-05927-y (PMC11401305; doi:10.1186/s12859-024-05927-y)
Supplement: Supplementary file 1 — Additional file 1: [file 12859_2024_5927_MOESM1_ESM.pdf]

scBubbletree: computational approach for visualization of single cell RNA-seq data — Supplementary material

This document includes:
- Supplementary Algorithm 1
- Supplementary Section 1-6
- Supplementary Figure 1-13

Supplementary Algorithms

Algorithm 1 Bubbletree generation.

Functions: min=minima of input values (function *min*, R-package base), sample=sampling with replacement (function *sample*, R-package base), HC=hierarchical clustering (function *hclust*, R-package stats), BBS=bootstrap branch support (function *prop.clades*, R-package ape)

Input:

k ▷ number of clusters

B ▷ number of bootstrap iterations

$C_1^{n_1\times f}, C_2^{n_2\times f}, \ldots, C_k^{n_k\times f}$ ▷ subsets of $A^{n\times f}$ for k clusters

$n_1, n_2, \ldots, n_k$ ▷ number of cells in each cluster

N_{eff} ▷ number of cells to draw from cluster

Output:

$\hat{H}$ ▷ consensus hierarchical dendrogram (bubbletree)

H_b ▷ bootstrap hierarchical dendrograms

for $b = 1$ to B **do**
  for $i = 1$ to $k-1$ **do**
    $n'_i \leftarrow \min(n_i, N_{eff})$
    $X^{n'_i\times f} \leftarrow \text{sample}(n'_i, C_i^{n_i\times f})$ ▷ draw n'_i rows from $C_i^{n_i\times f}$
    for $j = i+1$ to k **do**
      $n'_j \leftarrow \min(n_j, N_{eff})$
      $Y^{n'_j\times f} \leftarrow \text{sample}(n'_j, C_j^{n_j\times f})$ ▷ draw n'_j rows from $C_j^{n_j\times f}$
      $D_b^{ij} \leftarrow \frac{1}{n'_i n'_j}\sum_{\substack{\forall \mathbf{x}\in X\\ \forall \mathbf{y}\in Y}}\sqrt{(\mathbf{x}-\mathbf{y})^2}$ ▷ mean inter-cluster Euclidean distance between cells (row vectors) of X and Y
    end for
  end for
  $H_b \leftarrow \text{HC}(D_b^{ij})$ ▷ hierarchical clustering with average link
end for
$\hat{D}^{ij} \leftarrow \frac{1}{B}\sum_{b=1}^{B} D_b^{ij}$ ▷ inter-cluster distance for $i = 1, \ldots, k-1$ and $j = i+1, \ldots, k$
$\hat{H} \leftarrow \text{HC}(\hat{D}^{ij})$ ▷ consensus hierarchical clustering with average link
$\hat{H} \leftarrow \text{BBS}(\hat{H}, H_b)$ ▷ bootstrap branch support

Supplementary Sections

1 Impact of clustering algorithm on biological cluster homogeneity

Quantitative comparison of the five clustering algorithms available as part of scBubbletree was performed with datasets A and B (Supplementary Section 2): k-means, Louvain (original, LMR, SMR), and, partially also Leiden.

k-means clustering was performed with $k = 5$ and $k = 24$ for dataset A and B, respectively. Clustering resolution parameter $r = 0.003$ and $r = 0.794$ were used as input for the GCD methods for clustering of dataset A and B, respectively. Leiden clustering failed for dataset B due to the large size of the dataset.

The clustering results were compared with the adjusted RAND index (ARI) (function *adj.rand.index*, R-package fossil, version 0.4) [Hubert and Arabie, 1985]. ARI is a measure of similarity between two clustering results, yielding values between +1 (concordant results) and -1 (discordant results).

All five clustering methods produced similar results for dataset A (Supplementary Fig. S11A), and ARI was close to 1 between each pair of methods. The four GCD methods generated identical results (pairwise ARI = 1). For dataset B we observed considerable differences between the clustering results of the five methods (Supplementary Fig. S11C). The two Louvain variants, LMR and SMR, had similar results and ARI = 0.91. We also observed high degree of similarity (ARI $\in [0.78, 0.82]$) between the original Louvain method and LMR and SMR. ARIs were smaller (ARI ≈ 0.70) between the GCD methods and k-means.

The clustering results were also compared based on their weighted Gini impurities (WGIs). The WGIs were computed using the cell line labels of dataset A and the PBMC subtype labels from annotation set *l1* and *l2* of dataset B. For dataset A we saw WGIs close to 0 (low impurity) for all five clustering methods (Supplementary Fig. S11B). The GCD methods yielded similar WGIs, which were slightly lower than that of k-means. For dataset B, the GCD methods had systematically lower WGIs than k-means in terms of both annotation sets (Supplementary Fig. S11D). While the original Louvain algorithm marginally outperformed LMR and SMR with *l1*, application of SLM and LMR resulted in more homogeneous clusters than the original Louvain algorithm with *l2*.

In summary, we saw that the GCD methods tend to generate more homogenous clustering results than k-means, while the different Louvain variants and Leiden generated comparable results. Other clustering algorithms implemented in e.g. PhenoGraph [Levine et al., 2015] or TooManyCells [Schwartz et al., 2020], were not included in the benchmarking analysis as they rely on GCD method for clustering and are likely produce comparable results as the GCD methods

implemented in Seurat.

2 Datasets and data processing

2.1 Dataset A

scRNA-seq dataset A contains a mixture of 3,918 cells from five human lung adenocarcinoma cell lines (HCC827, H1975, A549, H838 and H2228). This dataset has been generated for the purposes of benchmarking of pipelines and methods for scRNA-seq analysis [Tian et al., 2019]. Sample collection and data processing details are provided in the respective publication [Tian et al., 2019]. Key data processing steps are summarized in the following. The single cell library were prepared using 10x Chromium platform and sequenced with Illumina NextSeq 500. Raw data was processed with Cellranger and analyzed by scPipe. demuxlet [Kang et al., 2018] was used to predict the most probable identity of each cell line based on the known genetic differences between the different cell lines. We obtained the dataset (with code *sc_10x_5cl*) and the cell type predictions from https://github.com/LuyiTian/sc_mixology.

2.2 Dataset B

scRNA-seq dataset B contains transcriptome data of 161,764 peripheral blood mononuclear cells (PBMCs) from eight healthy volunteers enrolled in a HIV vaccine trial [Hao et al., 2021]. Sample collection and data processing details are provided in the publication [Hao et al., 2021] and are summarized in the following. Samples were collected at three time points: immediately before (day 0), 3 days, and 7 days following administration of a HIV vaccine. All samples have been profiled using 10x Chromium protocol, followed by Illumina NovaSeq 6000 sequencing. Alongside the single cell transcriptomes, CITE-seq technology was used with up to 228 antibodies to profile the expression of cell-surface proteins. This multimodal data was processed with a weighted nearest neighbor (WNN) procedure to identify different cellular states in PBMCs. Cell types were predicted by this approach for each cell at two levels of resolution: *l1* with 8 cell types and *l2* with 31 cell types. The predictions are available as part of the meta data associated with the raw scRNA-seq data.

2.3 Data processing

Data processing of dataset A and B was performed with R-package Seurat (version 4.1.0). Normalization of gene expressions was done with the function *SCTransform* [Hafemeister and Satija, 2019] using default parameters, and principal

component analysis (PCA) was performed with function *RunPCA* based on the 5,000 most variable genes in the dataset identified with the function *FindVariableFeatures*.

In both datasets we saw that the first 15 principal components capture most of the variance in the data, and the proportion of variance explained by each subsequent principal component was negligible (Supplementary Fig. S1). Thus, we used the single cell projections (embeddings) in 15-dimensional feature space, $A^{3,918\times 15}$ and $A^{161,764\times 15}$, as input of scBubbletree. Dimensionality reduction of $A^{3,918\times 15}$ and $A^{161,764\times 15}$ to 2-dimensions (2D) by UMAP and t-SNE was performed with Seurat's functions *RunUMAP* and *RunTSNE*, respectively, using default parameters.

2.4 Quality control with scBubbletree

The quantitative nature of its output lends scBubbletree to applications not only to research but also to quality control. In the following we demonstrate this by quality control checks of datasets A and B.

2.4.1 Dataset A

The quality control of dataset A is summarized in the respective study [Tian et al., 2019]. We used scBubbletree to evaluate the relative frequency of mitochondrial RNA in the different bubbles (Supplementary Fig. S10C), which is an indicator of poor sample quality associated with high fraction of apoptotic or lysing cells. On average, about 19% percent of the RNA molecules originated from mitochondrial genes. We also observed bimodal distributions of the number of detected genes and RNA molecules in bubbles 0, 1 and 2 (Supplementary Fig. S10D-E). Nevertheless, the numbers of detected genes and RNA molecules were sufficiently high for downstream analysis.

2.4.2 Dataset B

Dataset B contains PBMC samples from 8 donors at 3 time points. Using donor and time used as categorical cell features, we visualized the relative frequency of cells from a specific donor and time across the different bubbles (Supplementary Fig. S5C). This allowed us to check for compositional biases, which is essential to e.g. identify novel cell types (e.g. tumor cells), or to detect systematic biases (e.g. batch effects) in a subset of the samples. This analysis revealed compositional biases in the samples of specific donors, for instance, all three samples of donors P2 and P3 had relatively higher frequency of bubble 0 and 2 cells, respectively.

To conduct a quantitative differential abundance analysis of cell type compositions [Buettner et al., 2021], scBubbletree provides tables with cell counts for each pair of sample and bubble.

As part of the quality checks we also looked for bubbles of cells with unusually high relative frequency of mitochondrial RNA (Supplementary Fig. S5F). We saw similar distributions of mitochondrial gene expression across the different bubbles, with about 95% of the cells having mitochondrial gene expression lower than 10%. In contrast to this, we saw differences in number of detected genes in the cells of the different bubbles. For instance, the cells in bubble 18 had lower numbers of detected genes (median number of genes $< 1{,}000$) and RNA molecules (median number of RNA molecules $< 2{,}500$) (Supplementary Fig. S5G-H). This may be explained by the fact that most cells in bubble 18 are classified as platelets. This cell type is devoid of nucleus and genomic DNA, and hence is incapable of de novo transcription [Melchinger et al., 2019].

3 Analysis of scRNA-seq samples from glioblastoma (GBM) patients

3.1 Dataset GBM

scRNA-seq dataset GBM contains transcriptome data of 45,466 cells from five human glioblastoma (GBM) patients [Schmassmann et al., 2023]. Paired samples were taken from three different tissues in each patient: tumor center (TC), peripheral infiltration zone (PI), and blood (PBMC). Sample collection and data processing details are provided in the publication [Schmassmann et al., 2023] and are summarized in the following. All samples have been profiled using 10X Genomics Chromium Single Cell Controller, followed by Illumina HiSeq 4000 sequencing. The raw data includes a barcode-gene count matrix and supplementary meta-data, such as cell types at different resolutions, cell phase, sample name, tissue site, QC metrics, and normalized gene expressions.

Data processing of the GBM dataset was performed with R-package Seurat (version 4.1.0). Normalization of gene expressions was done with the function *NormalizeData* using default parameters, and principal component analysis (PCA) was performed with the function *RunPCA* based on the 5,000 most variable genes in the dataset identified with the function *FindVariableFeatures*. We saw that the first 15 principal components capture most of the variance in the data, and the proportion of variance explained by each subsequent principal component was negligible. Thus, we used the single cell projections (embeddings) in 15-dimensional feature space, $A^{45{,}466 \times 15}$ as input of scBubbletree. The 2D t-SNE coordinates of each cell are provided in the metadata.

3.2 Determining the clustering resolution

To determine the clustering resolution, we computed the gap statistic for the Louvain method with resolution parameter $r = 10^R$, where R was initialized using a sequence of values from -4 to 1 in steps of 0.1 (Supplementary Fig. S12A). At each resolution we recorded the number k' of identified communities (Supplementary Fig. S12B).

The gap increased rapidly between $k' = 1$ and $k' = 13$, followed by a dip at $k' = 14$ to a value below 4. Between $k' = 13$ and $k' = 25$, the gap increased gradually by only about 0.15, even though 12 communities (about two-fold more) were added in this interval. This suggested that there is little benefit in using values of r larger than 0.158 ($k' = 13$). At higher k' we observed a slower and approximately linear increase in the gap.

The value of $k' = 13$ is close to the number of clusters identified in the publication [Schmassmann et al., 2023], which reports between $k = 10$ and $k = 22$ clusters of transcriptionally similar cells.

3.3 Clustering and hierarchical grouping of bubbles

For clustering of dataset GBM we chose the original Louvain method and a resolution parameter $r = 0.158$ ($k' = 13$). First, we constructed an SNN graph based on $A^{45,466 \times 15}$, where each vertex (cell) in the SNN graph was connected to its 50 nearest neighbors. Clustering was performed with 20 random starts and up to 100 iterations in each run. The clusters were organized in a bubbletree using hierarchical clustering with average linkage (Supplementary Fig. S12C). For this we used $B = 1,000$ bootstrap iterations and drew samples with up to $N_{eff} = 100$ cells from each cluster to compute inter-cluster distances.

The resulting bubbletree (Supplementary Fig. S12C) had 13 bubbles with sizes ranging from about 11,800 cells (26%, cluster 0) down to 184 cells (0.4%, cluster 12). Nearly all branches in the bubbletree were completely robust. The branch between bubbles 2 and 3 had low branch support as it was found in only 457 out of 1,000 bootstrap dendrograms.

3.4 Bubbletree evaluation

To determine which cell types are enriched in each bubble, we visualized the within-bubble relative frequencies of fine- and coarse-grained cell type labels (heatmaps in Supplementary Fig. S13A-B). The analysis showed that more than 95% of the cells in bubbles 8, 5, 11, 10, and 12 were CD45- (non-hematopoietic cells), while the remaining bubbles were enriched with immune cells, consistent with the results reported in [Schmassmann et al., 2023].

The bubbletree conserved global transcriptional distances between bubbles, grouping them into a PBMC clade with T-cells (bubbles 3, 2, and 1), B-cells (bubble 7), NK-cells (bubbles 3 and 2), monocytes (bubbles 9, 0, and 4), and neutrophils (bubbles 9 and 4). Additionally, it identified multiple non-PBMC populations including microglia (bubble 6), macrophages (bubble 4), tumor cells (bubbles 5 and 8), and other smaller cell populations.

Dataset GBM contains samples from three tissues: PBMC, tumor center, and peripheral infiltration zone. Using tissue as categorical cell features, we visualized the relative frequency of cells from a specific tissue within the different bubbles (heatmap in Supplementary Fig. S13C). We observed that many bubbles enriched with PBMC cell types (bubbles 2, 1, 7, 9, 0, and 4 in Supplementary Fig. S13A-B) had high relative frequency of cells from PBMC tissue (Supplementary Fig. S13C), except for bubble 3, which contained a mixture of cells from the tumor center and peripheral infiltration zone tissues. Hence, bubble 3 may contain tumor-infiltrating lymphocytes. The remaining bubbles in the bubbletree contained a mixture of cells from tumor center and peripheral infiltration zone tissues. Meanwhile, bubbles 5 and 8 were mostly composed of tumor cells from tumor centers.

Furthermore, dataset GBM contains samples from five patients and three tissues. Using tissue and patient as categorical cell features, we visualized the relative frequency of cells from a specific tissue and patient across the different bubbles (heatmap in Supplementary Fig. S13D) as well as within the bubbles (heatmap in Supplementary Fig. S13E). This allowed us to check for compositional biases, which is essential to e.g. identify novel cell types (e.g. tumor cells), or to detect systematic biases (e.g. batch effects) in a subset of the samples. In the next, we describe our key findings. First, all PBMC samples (middle columns Supplementary Fig. S13D) had similar relative abundances across the bubbles. In contrast, the samples from tumor center and peripheral infiltration zone were heterogeneous in terms of their relative abundances across the bubbles. Second, all bubble 5 cells came from patient 604 (95.6% from center-604 and 4.4% periphery-604) (Supplementary Fig. S13E), and 69.4% of the cells in sample center-604 were assigned to bubble 5 (NPC-like tumor cells) (Supplementary Fig. S13D). Third, the majority of cells in bubbles 11, 10, and 12 came from patients 604 and 617 (Supplementary Fig. S13E).

Additional evaluation of the bubbletree was done by visualizing the relative frequency of cell phases within bubbles (heatmap in Supplementary Fig. S13F), or the normalized expression of 4 marker genes (panels in Supplementary Fig. S13G) in cells (x-axis) from each bubble. These results were consistent with the results reported in [Schmassmann et al., 2023]. Note that Supplementary Fig. S13 demonstrates that bubbletrees can be easily and coherently stacked with additional quantitative information, thus facilitating biological interpretation.

A more detailed description of the workflow and the source code is available at: https://github.com/snaketron/scBubbletree_paper_data

4 Gene expression of human cancer cell lines

We obtained gene expression data for 1,019 human cancer cell lines from the Cancer Cell Line Encyclopedia [Barretina et al., 2012] (https://www.ebi.ac.uk/gxa/-experiments/E-MTAB-2770/Results, obtained on 20.04.2022), and selected among them 69 cell lines that contained the keyword "lung adenocarcinoma" in their names. Expressions (transcript per million; TPM) were available for 56,443 genes. To quantify the distance in gene expression between a pair of cell lines, we computed the Euclidean distance between the corresponding gene expression vectors (function *dist*, R-package stats, version 4.2). This procedure was repeated for 2,346 pairs of cell lines. In the distribution of Euclidean distances between the cell lines we saw a conspicuously small distance between cell lines H1975 and HCC827 (Supplementary Fig. S3). The distances between the remaining cell lines were closer to the mean of the distribution.

5 Evaluation of the bubbletree of dataset B based on annotation set *l2*

The composition of the bubbles was also investigated based on the more detailed annotation set *l2*. Using the function *get_cat_tiles* we visualized the within-bubble relative frequencies of 31 PBMC subtypes from *l2* (heatmap in Supplementary Fig. S5B) and made the following observations. First, the bubbles of clade 'b' were enriched with cells from distinct populations of monocytes and DCs (Supplementary Fig. S5B). Bubbles 1, 3 and 5, which mostly contained $CD14^{+}$ classical monocytes, were characterized by high CD14 and low CD16 protein and RNA expression (RNA heatmap in Supplementary Fig. S5D-E). About 74% of all monocytes were found in these bubbles (Supplementary Fig. S5A), and this was comparable to the expected relative frequency of classical monocytes in humans [Patel et al., 2017]. Bubbles 12 and 19 contained a mixture of $CD14^{+}$ classical monocytes and $CD16^{+}$ non-classical monocytes. As CD14 and CD16 were both highly expressed in bubble 12 and bubble 19 cells, it is likely that these bubble are enriched with intermediate monocytes. Interestingly, bubble 19 was also enriched with cells characterized as doublets (Supplementary Fig. S5B). Finally, bubble 13 was enriched with $CD16^{+}$ non-classical monocytes, whereas bubble 16 contained a mixture of $CD14^{+}$ classical monocytes and three populations of DCs: conventional DC1 and DC2 (cDC1 and cDC2) and Axl^{+} $Siglec6^{+}$ DC (ASDC).

Second, the bubbles of the B cell subclade were enriched with functionally distinct subtypes of B cell, i.e. bubble 10 contained mainly naive B cells (98% of the bubble cells), bubble 11 contained a mixture of intermediate (39% of the bubble cells), memory (55% of the bubble cells) and a small fraction of naive B cells (5% of the bubble cells), while bubble 23 was enriched with plasmablasts (98% of the bubble cells). The small bubbles 21 and 22 contained proliferating lymphocytes and hematopoietic stem and progenitor cell (HSPC), respectively.

Bubbles 2 and 15 made up the NK subclade and were enriched with cells from two functionally different NK subtypes that are characterized by distinct expression of the surface marker CD56 (ADT heatmap in Supplementary Fig. S5E): $CD56^{bright}$ and $CD56^{dim}$. In bubble 2 we saw an enrichment of $CD56^{dim}$ NK cells (99% of the bubble cells), and bubble 15 contained a mixture of $CD56^{dim}$ and $CD56^{bright}$ NK cells (69% and 27% of the bubble cells, respectively).

Third, the remaining bubbles of clade 'a' were enriched with cells from different T cell subtypes. Functionally and/or developmentally related subtypes of T cells were found in common subclades, for instance, bubble 6 was enriched with naive $CD8^+$ T cells (97% of the bubble cells), while bubble 0 was enriched with naive $CD4^+$ T cells (87% of the bubble cells). These two bubbles accounted for about 18% of all cells in the sample and about 50% of all T cells. Similarly, bubble 4 and 7 were primarily enriched with different memory subtypes of $CD4^+$ and $CD8^+$ T cells. Bubbles 9 and 14 were enriched with $CD8^+$ memory T cells, $CD8^+$ effector memory T cells (CD8 TEM), mucosal associated invariant (MAIT) T cells and $\gamma\delta$ T cells, whereas the bubbles 8 and 17 were enriched with $CD4^+$ T cells with cytotoxic activity (CD4 CTL), $CD8^+$ effector memory T cells (CD8 TEM) and T cells which share phenotype with NK cells and $\gamma\delta$ T cells.

Fourth, the outgroup bubble 18 was enriched with platelets (99% of cells in that bubble), whereas bubble 20 contained plasmacytoid dendritic cells (pDCs, 96% of cells in bubble) and a small fraction of ASDCs (4% of the cells in bubble).

6 Quantifying uncertainty with Highest Density Intervals (HDIs)

The HDI was used to summarize the most credible points of a distribution (function *hdi*, R-package bayestestR, version 0.13). All points within the HDI have a higher probability density than points outside the interval, and the total mass of points inside the 95% HDI is 95% of the distribution.

Supplementary Figures

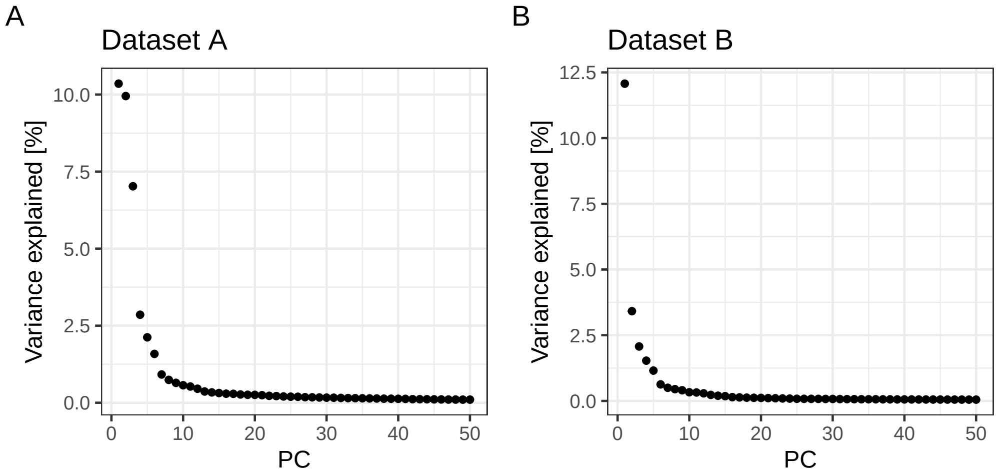

Supplementary Figure S1. Percent of variance explained by principal components 1 to 50 in data set A (panel A) and B (panel B).

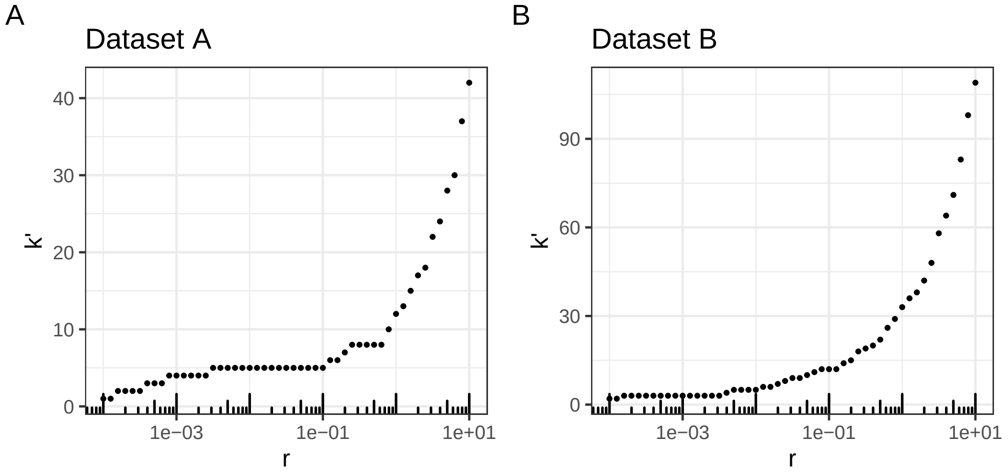

Supplementary Figure S2. Relationship between resolution (r) and the number of identified communities (k') by Louvain clustering of dataset A (panel A) and dataset B (panel B).

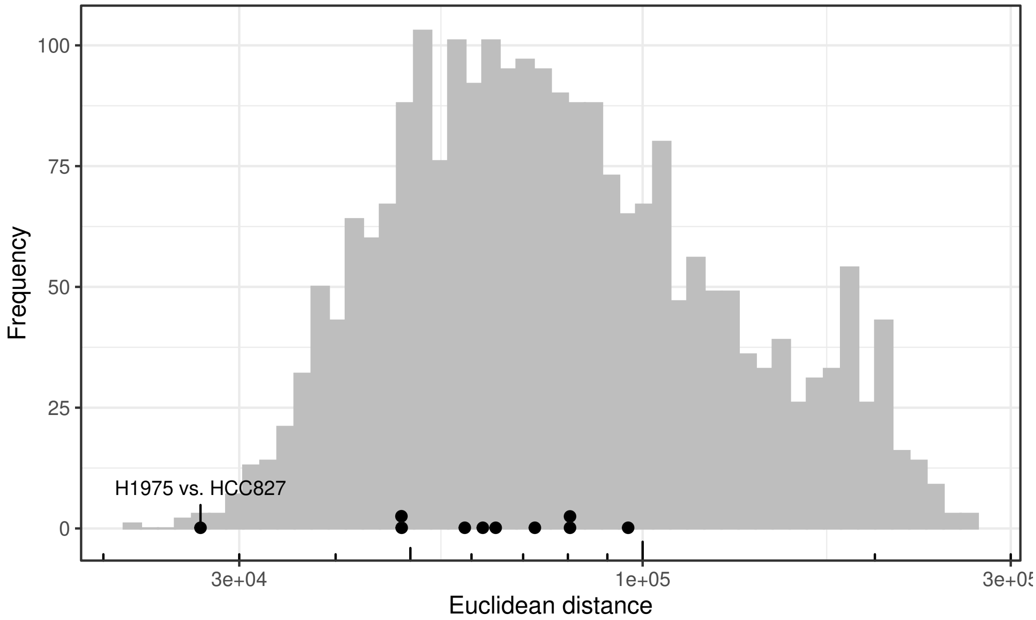

Supplementary Figure S3. Histogram of distances in gene expression between 2,346 pairs of 69 human lung adenocarcinoma cell lines. Distances between cell lines H838, H2228, A549, H1975 and HCC827 are shown with black filled points. The distance between cell lines H1975 and HCC827 is annotated with a black label. Vertical jitter was added to avoid overplotting of black points.

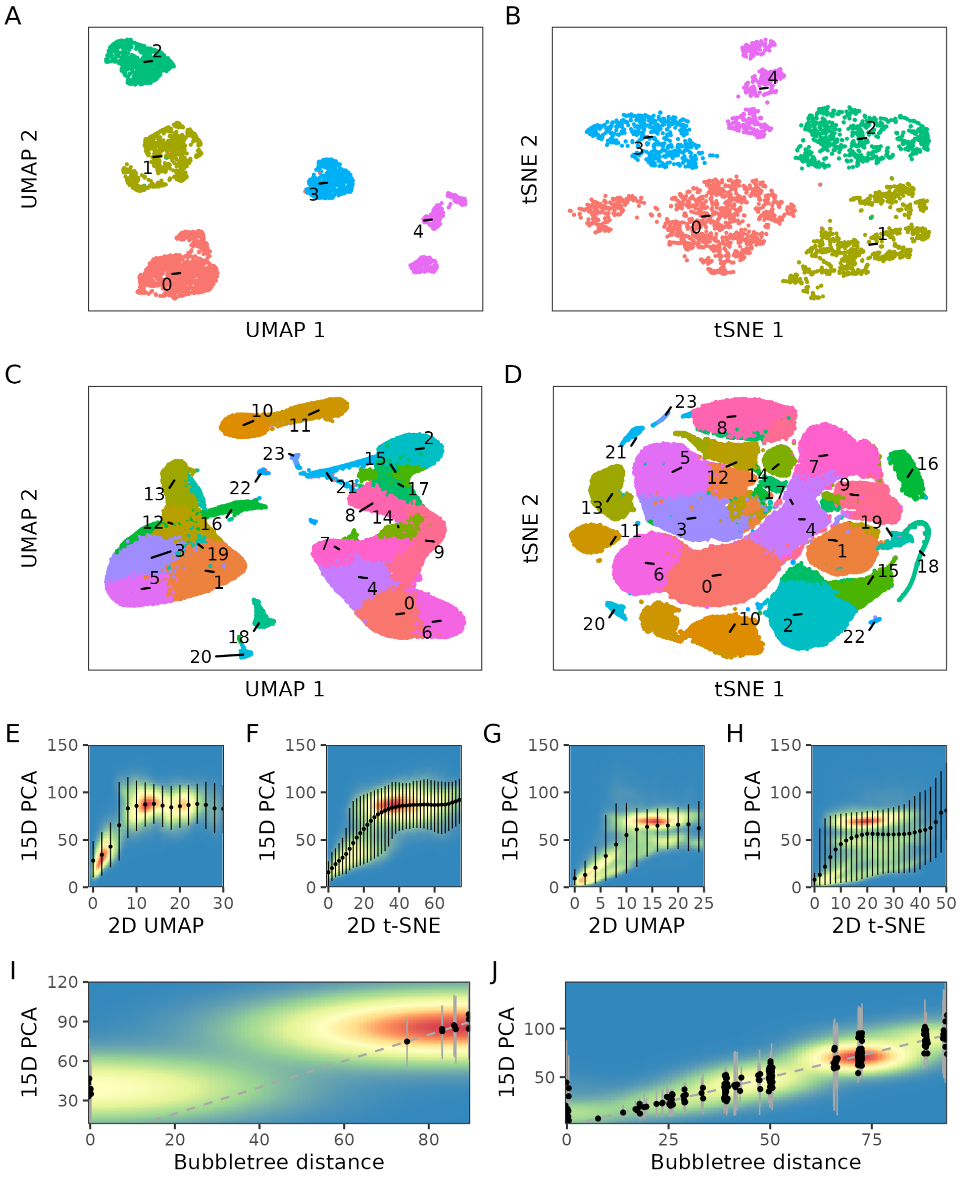

Supplementary Figure S4. 2D UMAP and t-SNE visualization and distance structure of dataset A and B. (A, B) 2D UMAP and t-SNE of dataset A. (C, D) 2D UMAP and t-SNE of dataset B. Filled points represent cells, color coded according to their bubble IDs. (E, F, G, H) 2D density of distances in 2D UMAP and t-SNE vs. 15D PCA space between 10^6 cell pairs, randomly selected with replacement from data sets A (panels E, F) and B (panels G, H). Blue, yellow and red density gradient color codes for low, medium and high density, respectively. Points are means and vertical error bars are 95% highest density intervals (HDIs, Supplementary Section 5) of the cell-cell distances in 15D PCA space, computed over the data of overlapping x-axis bins obtained by sliding window approach (sliding step = 2, window length = 4). (I, J) 2D density of distances between bubbles in the bubbletree (x-axis, with random jitter of points and error bars along the horizontal axis to reduce overplotting) and average Euclidean distances between cells in 15D PCA space (y-axis) for dataset A (panel I) and B (panel J). Error bars are 95% HDIs of the distances in 15D PCA space between 10^4 pairs of cells randomly selected with replacement from each pair of bubbles. Blue, yellow and red density gradient color codes for low, medium and high density, respectively. Dashed gray lines are diagonals.

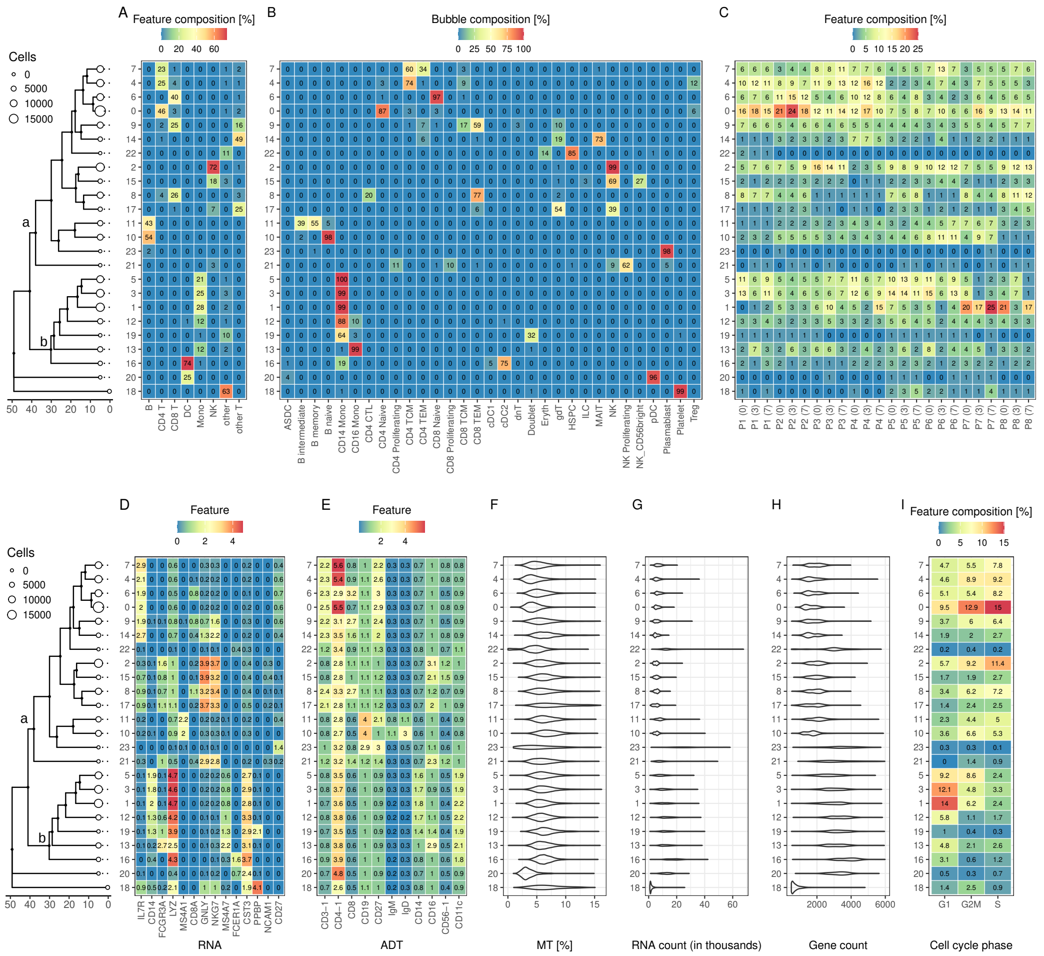

Supplementary Figure S5. Visualization of categorical and numeric features of dataset B. A simplified version of the bubbletree from Fig. 2C is appended to the two panel rows. (A) Across-bubble relative frequencies (%) of cell types (*l1*; x-axis) rounded to the nearest integer. Columns integrate to 100% (up to rounding error). (B) Within-bubble relative frequencies (%) of cell types (*l2*; x-axis) rounded to the nearest integer. Rows integrate to 100% (up to rounding error). (C) Across-bubble (y-axis) relative frequencies of sample names (x-axis) rounded to the nearest integer. Columns integrate to 100% (up to rounding error). Samples names are derived from a combination of donor IDs (P1-P8) and time points (0, 3 and 7). (D) Mean normalized expression (RNA) of 14 genes (x-axis) in each bubble (y-axis). (E) Mean normalized expression (ADT) of 11 surface-proteins (x-axis) in each bubble (y-axis). (F-H) Quality control of dataset B. Violins show in different bubbles (y-axis) the distribution of (F) the relative contribution to the total gene expression in a cell coming from mitochondrial genes, (G) the number of detected RNA molecules, and (H) the number of detected genes. (I) Across-bubble (y-axis) relative frequencies of cell cycle phase labels (x-axis) rounded to the nearest one. Columns integrate to 100% (up to rounding error).

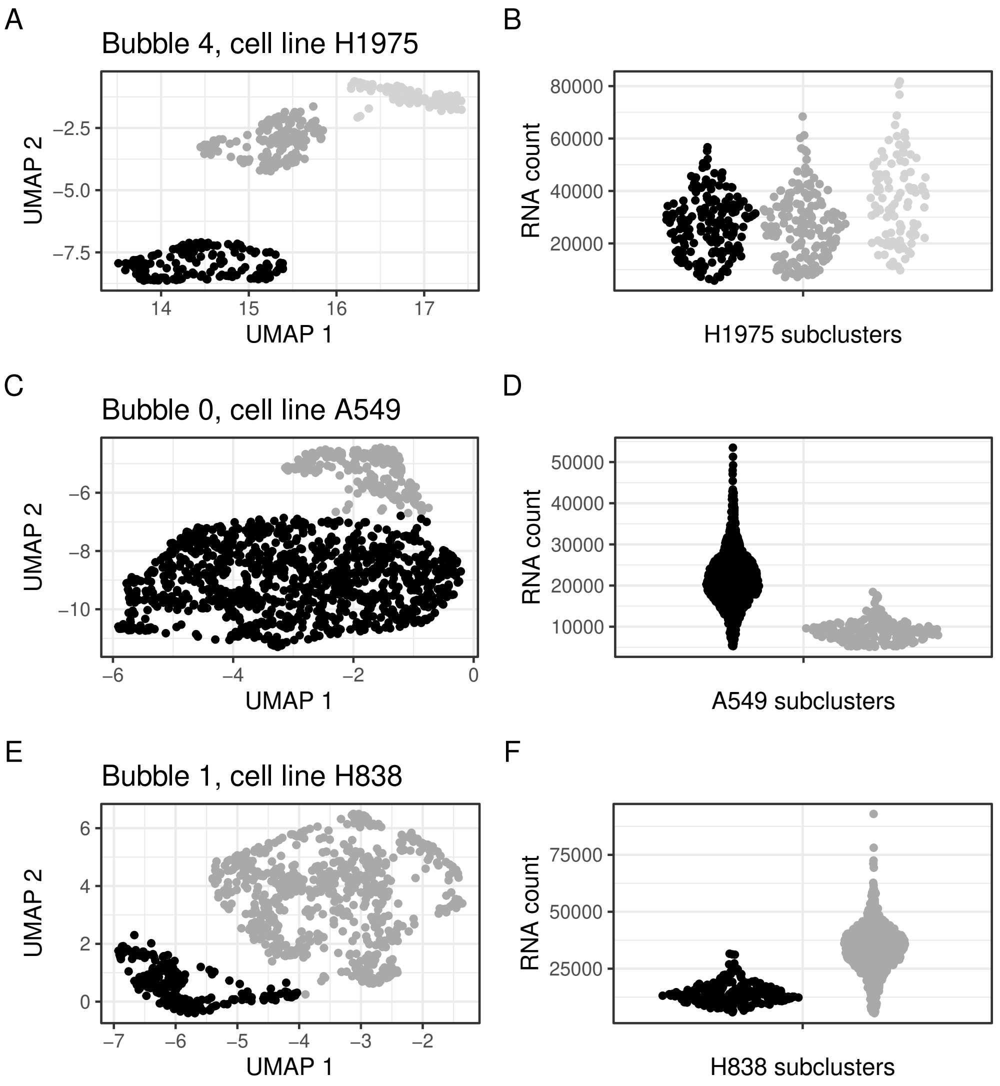

Supplementary Figure S6. Cell line subclusters in 2D UMAP space of dataset A. (A, C, E) Cells in different subclusters (indicated by different shades of gray) in bubble 4 (panel A), bubble 0 (panel C) and bubble 1 (panel E). Cells from bubble 4, 0 and 1 are enriched with cells from cell line H1975, A549 and H838, respectively. (B, D, F) Distribution of the number of RNA molecules detected in cells from each subcluster of bubble 4 (panel B), 0 (panel D) and 1 (panel F).

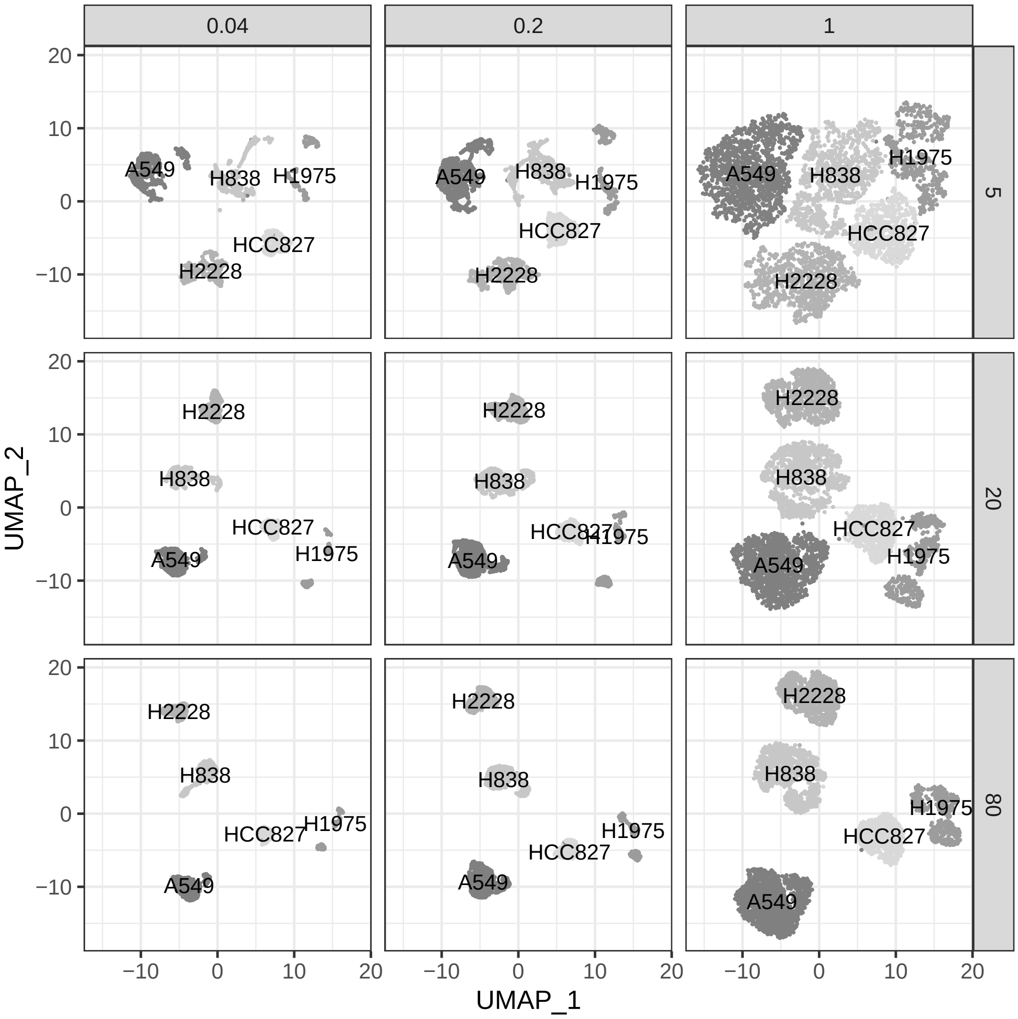

Supplementary Figure S7. Effects of UMAP hyperparameters *min_dist* and *n_neighbors* on the 2D UMAP embeddings of dataset A. Filled points are cells, gray-level coded according to their cell lines. Column panels correspond to *min_dist*=0.04, 0.02 and 1; row panels correspond to *n_neighbors*=5, 20 and 80. Default values were used for the remaining two UMAP hyperparameters, *d*=2 and *n_epoch*=300. UMAP dimensionality reduction was performed with the function *RunUMAP* from the R-package Seurat. Visualization was performed with the R-package ggplot2. Parameter legend: *min_dist*: the neighborhood size to use for local metric approximation; *n_neighbors*: parameter for controlling the layout; *n_epoch*: the number of training epochs to be used in optimizing the low dimensional embedding; *d*: the number of dimensions.

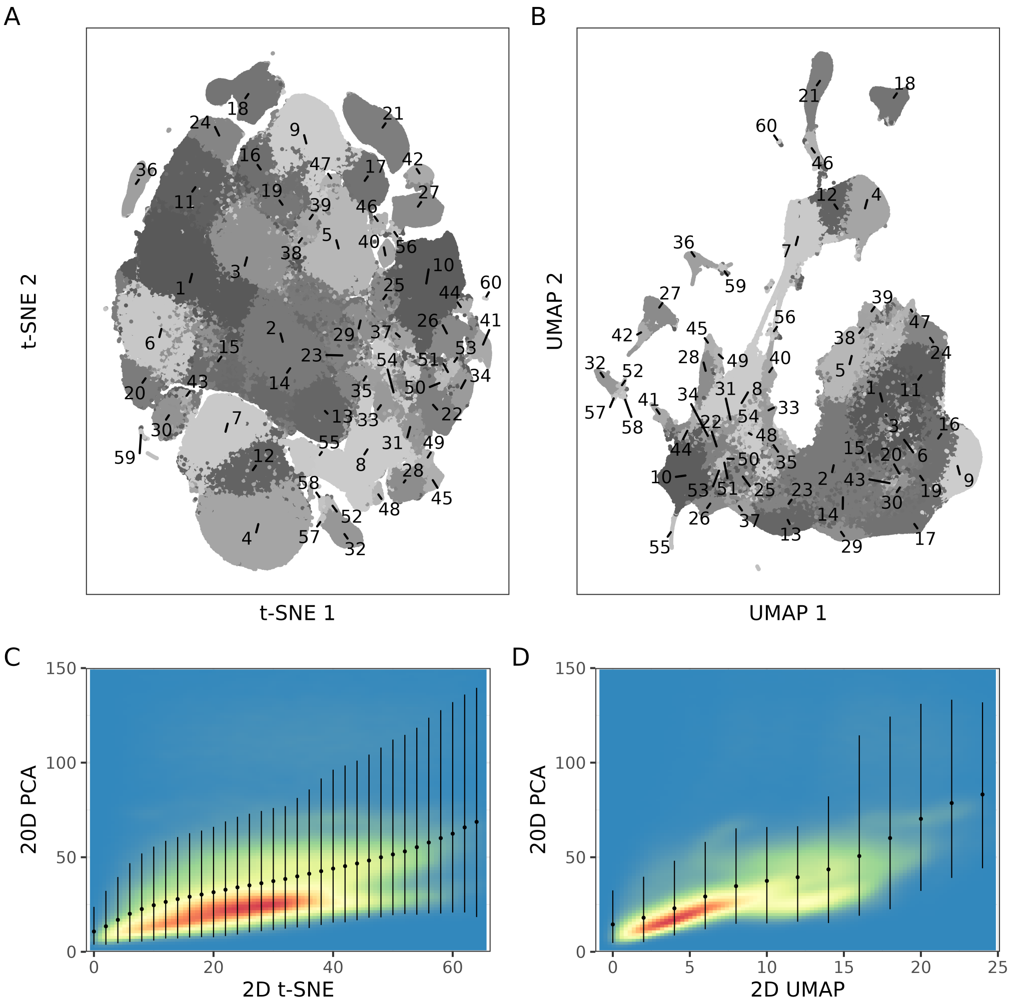

Supplementary Figure S8. 2D t-SNE and UMAP and distance structure of scRNA-seq data of 1.3 million mouse brain cells. (A) 2D t-SNE plot. Filled dots are cells, grey-level coded according to their cluster IDs. Labels show cluster IDs. PCA, t-SNE and graph-based clustering data were downloaded from https://support.10xgenomics.com/single-cell-gene-expression/datasets/1.3.0/1M_neurons? on 26.10.2022. (B) UMAP plot generated with function *umap* (R-package umap, version 0.2.10). Filled dots are cells color coded according to their cluster IDs. Labels show cluster IDs. (C, D) 2D density of distances between cells in 2D t-SNE (panel C) and 2D UMAP (panel D) vs. 20D PCA space between 10^6 cell pairs randomly selected with replacement. Blue to red gradient colors reflect low to high density. Points are means and vertical error bars are 95% highest density intervals (HDIs, Supplementary Section 5) of the cell-cell distances in 20D PCA space, computed over the data of overlapping x-axis bins obtained by sliding window approach (sliding step=2, window length=4).

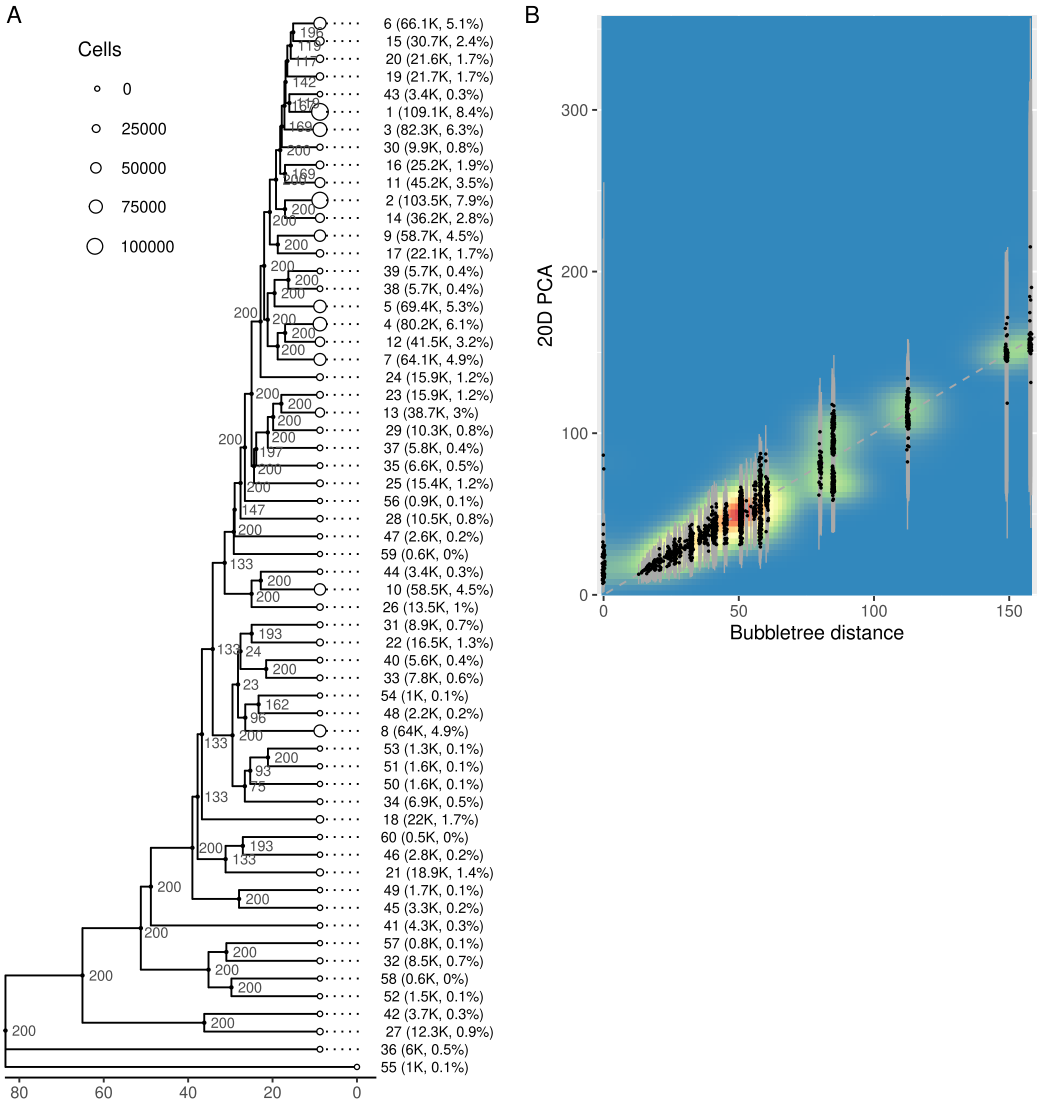

Supplementary Figure S9. bubbletree of scRNA-seq data of 1.3 million mouse brain cells. (A) Bubbletree generated with function *get_bubbletree_dummy* based on 20D PCA data and cluster assignments. We performed $B = 200$ bootstrap iterations and drew samples with up to $N_{eff} = 400$ cells from each cluster to estimate inter-cluster distances. PCA and graph-based clustering data were downloaded from https://support.10xgenomics.com/single-cell-gene-expression/datasets/1.3.0/1M_neurons? on 26.10.2022. (B) 2D density of distances between bubbles in the bubbletree (x-axis, with random jitter along the horizontal axis to reduce overplotting) and average Euclidean distances between cells in 20D PCA space (y-axis). Error bars are 95% highest density intervals (HDIs, Supplementary Section 5) of the distances in 20D PCA space between 10^4 pairs of cells randomly selected with replacement from each pair of bubbles. Blue, yellow and red density gradient color codes for low, medium and high density, respectively. Diagonal is shown as dashed gray line.

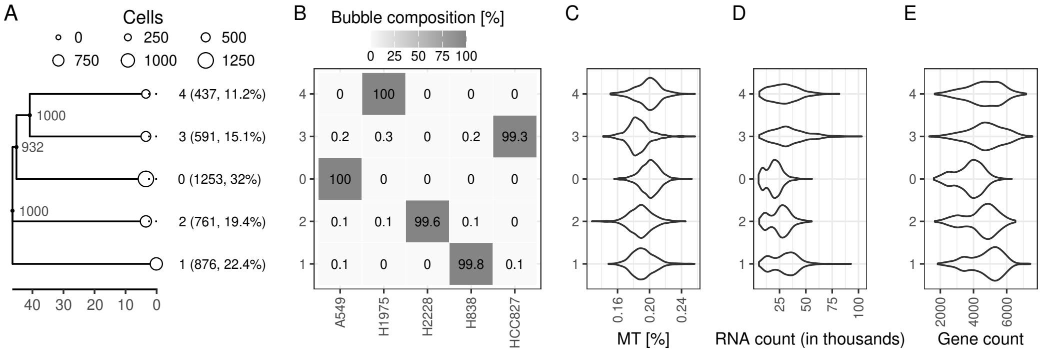

Supplementary Figure S10. Bubbletree and quality control of dataset A. (A) bubbletree annotated with a (B) heatmap displaying as rows the within-bubble relative frequencies of different cell lines. The bubbletree has five bubbles (white points) shown as leaves. Bubble radii scale linearly with the number of cells in the bubbles. Bubble identities, absolute and relative cell frequencies are shown as labels between bubbletree and heatmap. Red labels are branch bootstrap support values. Rows in the heatmap integrate to 100%. Violins show in different bubbles (y-axis) the distribution of (C) the relative contribution to the total gene expression in a cell coming from mitochondrial (MT) genes, (D) the number of detected RNA molecules and (E) the number of detected genes.

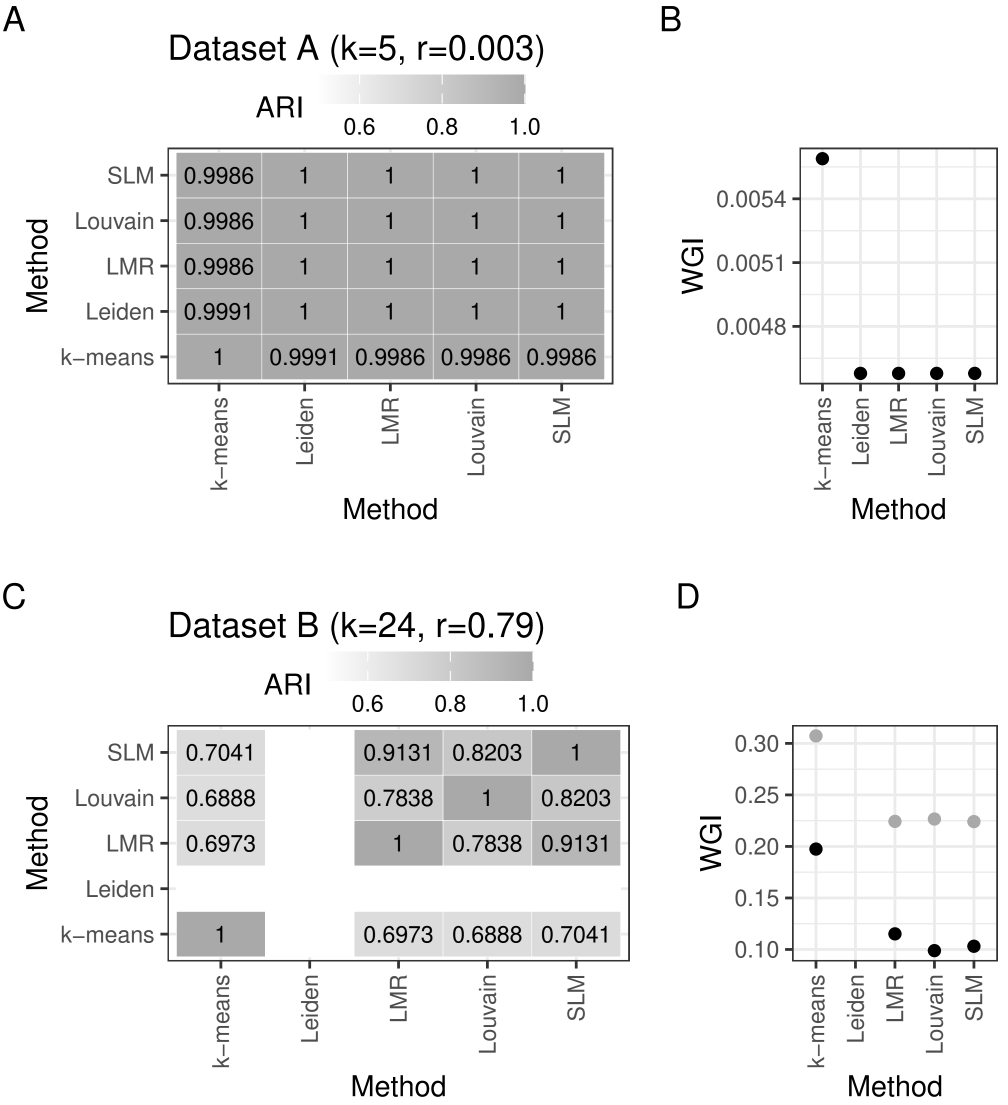

Supplementary Figure S11. Comparison of clustering algorithms. (A, C) Tiles are gray-level coded and labeled according to the adjusted RAND index (ARI) between pairs of clustering solutions from five clustering algorithms implemented in scBubbletree. ARIs are shown for dataset A (panel A) and dataset B (panel C). Clustering resolution parameter $k = 5$ and $k = 24$ were used as input for k-means for clustering of dataset A and B, respectively. Clustering resolution parameter $r = 0.003$ and $r = 0.794$ were used as input for the graph-based community detection methods for clustering of dataset A and B, respectively. Leiden clustering failed for dataset B due to the large size of the dataset. (B) Points are weighted Gini impurity (WGI) indices for the five clustering algorithms and the cell line labels of dataset A. (D) Points are WGIs for the five clustering algorithms and the PBMC subtype labels from annotation set *l1* and *l2* of dataset B. Louvain: original Louvain algorithm, LMR: Louvain algorithm with multilevel refinement, SLM: smart local moving, Leiden: Leiden algorithm.

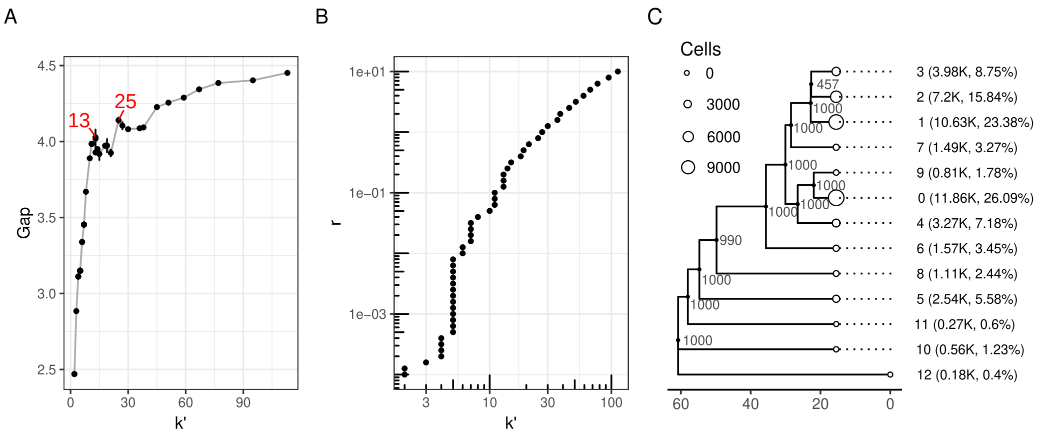

Supplementary Figure S12. scBubbletree analysis of dataset GBM. (A) Gap statistic for clustering solutions generated by the Louvain method with varying clustering resolutions. (B) Relationship between resolution (r) and the number of identified communities (k') by Louvain clustering. (C) bubbletree (tree structure) with 13 bubbles.

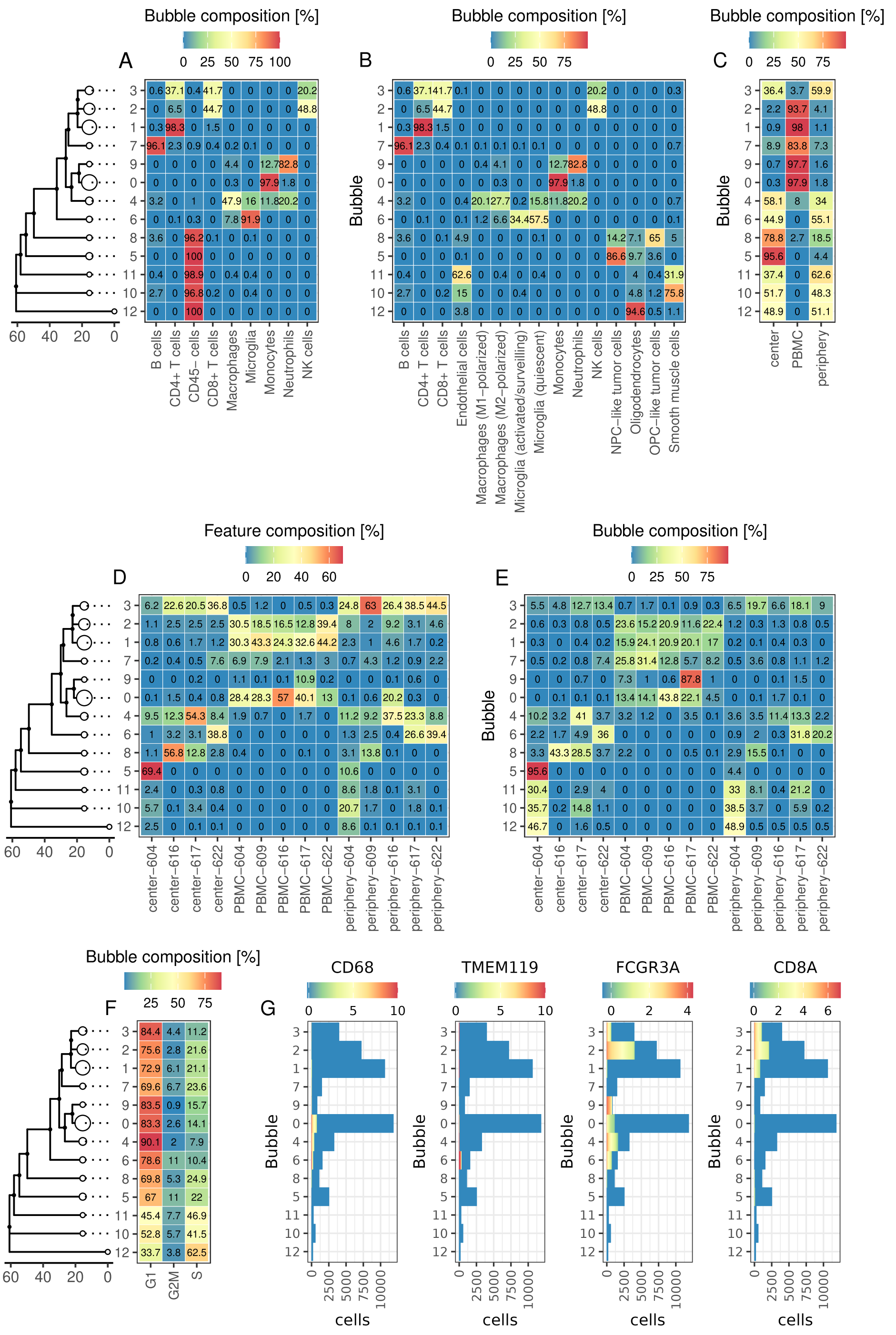

Supplementary Figure S13. Visualization of cell features of dataset GBM. A simplified version of the bubbletree from Supplementary Fig. S12C is appended to the three panel rows. (A-B) Within-bubble (y-axis) relative frequencies (%) of fine- (A) and coarse-grained (B) cell types (x-axis). Rows integrate to 100% (up to rounding error). (C) Within-bubble (y-axis) relative frequencies of tissue labels (x-axis). Rows integrate to 100%. (D) Across-bubble (y-axis) relative frequencies sample labels (x-axis). Columns integrate to 100%. (E) Within-bubble (y-axis) relative frequencies sample labels (x-axis). Rows integrate to 100%. Samples labels are derived from a combination of tissue and donor ID. (F) Within-bubble (y-axis) relative frequencies of cell cycle phase labels (x-axis). Rows integrate to 100%. (G) Normalized expression (blue, yellow and red gradient color codes for low, medium and high expression, respectively) of 4 genes (shown as panels) in cells (x-axis) from each bubble (y-axis).

References

Jordi Barretina, Giordano Caponigro, Nicolas Stransky, Kavitha Venkatesan, Adam A Margolin, Sungjoon Kim, Christopher J Wilson, Joseph Lehár, Gregory V Kryukov, Dmitriy Sonkin, et al. The cancer cell line encyclopedia enables predictive modelling of anticancer drug sensitivity. *Nature*, 483(7391): 603–607, 2012.

Maren Buettner, Johannes Ostner, Christian L Mueller, Fabian J Theis, and Benjamin Schubert. sccoda is a bayesian model for compositional single-cell data analysis. *Nature communications*, 12(1):1–10, 2021.

Christoph Hafemeister and Rahul Satija. Normalization and variance stabilization of single-cell rna-seq data using regularized negative binomial regression. *Genome biology*, 20(1):1–15, 2019.

Yuhan Hao, Stephanie Hao, Erica Andersen-Nissen, William M Mauck III, Shiwei Zheng, Andrew Butler, Maddie J Lee, Aaron J Wilk, Charlotte Darby, Michael Zager, et al. Integrated analysis of multimodal single-cell data. *Cell*, 184(13): 3573–3587, 2021.

Lawrence Hubert and Phipps Arabie. Comparing partitions. *Journal of classification*, 2:193–218, 1985.

Hyun Min Kang, Meena Subramaniam, Sasha Targ, Michelle Nguyen, Lenka Maliskova, Elizabeth McCarthy, Eunice Wan, Simon Wong, Lauren Byrnes, Cristina M Lanata, et al. Multiplexed droplet single-cell rna-sequencing using natural genetic variation. *Nature biotechnology*, 36(1):89–94, 2018.

Jacob H Levine, Erin F Simonds, Sean C Bendall, Kara L Davis, D Amir El-ad, Michelle D Tadmor, Oren Litvin, Harris G Fienberg, Astraea Jager, Eli R Zunder, et al. Data-driven phenotypic dissection of AML reveals progenitor-like cells that correlate with prognosis. *Cell*, 162(1):184–197, 2015.

Hannah Melchinger, Kanika Jain, Tarun Tyagi, and John Hwa. Role of platelet mitochondria: life in a nucleus-free zone. *Frontiers in cardiovascular medicine*, 6:153, 2019.

Amit A Patel, Yan Zhang, James N Fullerton, Lies Boelen, Anthony Rongvaux, Alexander A Maini, Venetia Bigley, Richard A Flavell, Derek W Gilroy, Becca Asquith, et al. The fate and lifespan of human monocyte subsets in steady state and systemic inflammation. *Journal of Experimental Medicine*, 214(7): 1913–1923, 2017.

Philip Schmassmann, Julien Roux, Steffen Dettling, Sabrina Hogan, Tala Shekarian, Tomás A Martins, Marie-Françoise Ritz, Sylvia Herter, Marina Bacac, and Gregor Hutter. Single-cell characterization of human gbm reveals regional differences in tumor-infiltrating leukocyte activation. *Elife*, 12:RP92678, 2023.

Gregory W Schwartz, Yeqiao Zhou, Jelena Petrovic, Maria Fasolino, Lanwei Xu, Sydney M Shaffer, Warren S Pear, Golnaz Vahedi, and Robert B Faryabi. TooManyCells identifies and visualizes relationships of single-cell clades. *Nature methods*, 17(4):405–413, 2020.

Luyi Tian, Xueyi Dong, Saskia Freytag, Kim-Anh Lê Cao, Shian Su, Abolfazl Jalal-Abadi, Daniela Amann-Zalcenstein, Tom S Weber, Azadeh Seidi, Jafar S Jabbari, et al. Benchmarking single cell RNA-sequencing analysis pipelines using mixture control experiments. *Nature methods*, 16(6):479–487, 2019.
